# Inferring immune‐associated signatures based on a co‐expression network in Guillain‐Barré syndrome

**DOI:** 10.1111/cpr.12634

**Published:** 2019-05-16

**Authors:** Xiaoming Zhang, Huixue Zhang, Zhaojun Liu, Ruoyu Guan, Jianjian Wang, Xiaotong Kong, Lixia Chen, Chunrui Bo, Jie Li, Ming Bai, Xiaoyu Lu, Jia Shen, Lihua Wang, Mian Guo

**Affiliations:** ^1^ Department of Neurology The Second Affiliated Hospital of Harbin Medical University Harbin Heilongjiang China; ^2^ Department of Neurosurgery The Second Affiliated Hospital of Harbin Medical University Harbin Heilongjiang China; ^3^ School of Dentistry University of California, Los Angeles Los Angeles California

**Keywords:** biomarker, co‐expression, Guillain‐Barré syndrome, immune, network

## Abstract

**Objectives:**

Guillain‐Barré syndrome (GBS) is a type of acute autoimmune disease, which occurs in peripheral nerves and their roots. There is extensive evidence that suggests many immune‐associated genes have essential roles in GBS. However, the associations between immune genes and GBS have not been sufficiently examined as most previous studies have only focused on individual genes rather than their entire interaction networks.

**Materials and methods:**

In this study, multiple levels of data including immune‐associated genes, GBS‐associated genes, protein‐protein interaction (PPI) networks and gene expression profiles were integrated, and an immune or GBS‐directed neighbour co‐expressed network (IOGDNC network) and a GBS‐directed neighbour co‐expressed network (GDNC network) were constructed.

**Results:**

Our analysis shows the immune‐associated genes are strongly related to GBS‐associated genes whether at the interaction level or gene expression level. Five immune‐associated modules were also identified which could distinguish between GBS and normal samples. In addition, functional analysis indicated that immune‐associated genes are essential in GBS.

**Conclusions:**

Overall, these results highlight a strong relationship between immune‐associated genes and GBS existed and provide a potential role for immune‐associated genes as novel diagnostic and therapeutic biomarkers for GBS.

## 1 INTRODUCTION

Guillain‐Barré syndrome (GBS) is a type of acute autoimmune disease, which occurs in the peripheral nerves and their roots.^1^, ^2^ A monophasic course and varying clinical outcomes are common characteristics of GBS.^3^ GBS is a serious disease which develops rapidly and could be life‐threatening during the acute phase. Patients usually suffer from morbus asthenicus, flexor weakness, sensory disorders and neurological symptoms.^4^ Weakness often involves respiratory muscles, making patients dependent on respirators.^5^ Patients with an infectious disease are more likely to develop GBS. At nerve membranes, the antibodies produced from the immune response can interact with gangliosides.^6^ Supportive medical care and immunotherapy are two main treatment modes for GBS. Although most patients should recover by nonspecific immunotherapy, there is still a need for optimized treatment and patient care throughout the course of disease.^7^ It is also essential to optimize supportive medical care to prevent or treat disease‐related complications. Therefore, there is an urgent need to identify novel biomarkers and treatment methods to improve the clinical features of GBS.

Guillain‐Barré syndrome has been proven to be related to immune dysfunction. A previous study reported that humoral immune and autoimmune responses could be triggered by pathogen infection (eg, Campylobacter jejuni) leading to nerve dysfunction for GBS.^8^ It has also been demonstrated that the activation of prominent T lymphocytes (T cells) occurs in GBS and is essential in the pathogenesis of a rat model of the disease. The integrins on T cells and adhesion molecules are up‐regulated on the endoneurial endothelium in GBS.^9^ Although some studies reported a relationship between the immune system and GBS, the roles of immune factors in the pathogenesis of GBS have not been explored in detail and systematically investigated.

Genetic factors are also important contributors for the pathogenesis of GBS. Others have reported associations between the development of GBS and CD1 SNPs in European populations,^10^ a finding also reported in the Chinese population.^11^ Yuan et.al suggested that CCR2 is responsive to pharmacologic blockade CCR2, making a candidate drug target for GBS which could provide novel insight into exploring target‐specific anti‐inflammatory treatment methods for peripheral neuroinflammation.^12^ Genome‐wide gene expression analyses of GBS suggest there are gender differences based on GBS patients.^13^ Chang et.al identified networks and key pathways associated with GBS using gene expression profiles.^14^ These studies implicate certain genes in the development of GBS, and gene expression profiles may help identify GBS‐associated pathways. However, most of these studies concentrated on individual genes and either used cell lines or limited patient samples. Specially, the global and systematic investigation focus on the association between immune and GBS using genome‐wide expression profiling is absent.

In the current study, an immune‐ or GBS‐directed neighbour co‐expressed network (IOGDNC network) including immune‐ and GBS‐associated genes, immune‐associated genes only, GBS‐associated genes only, and other genes was constructed. We also extracted the first neighbours of GBS‐associated genes from IOGDNC networks to construct a GBS‐directed neighbour co‐expressed network (GDNC network). We use the two networks to illustrate the roles of immune‐associated genes in GBS. We identified immune‐associated genes in the IOGDNC network with hub topological characteristics related to GBS. The immune‐ and GBS‐associated genes have the highest co‐expression level are also found. We further identify five clusters from GDNC network which include some key immune‐associated and GBS‐associated genes, suggesting specific functions of the immune‐associated genes in GBS. Specially, some of these clusters could distinguish the GBS from control samples when used as a ebiomarker. A functional analysis reveals that the genes in these clusters are co‐related to immune‐related processes and others in GBS. Our results highlight the specific contribution of an immune‐directed network for GBS and could be as an effective resources and candidate for further research and treatment of GBS.

## 2 MATERIALS AND METHODS

### 2.1 Expression profile of genes and PPI network data set

High‐throughput microarray gene expression data were obtained from Gene expression Omnibus (GEO, accession number: GSE31014). This data set comprises 7 Guillain‐Barré syndrome samples and 7 normal controls. We downloaded high‐confidence protein‐protein interaction (PPI) data from the Human Protein Reference Database (HPRD),^15^ which is an integrated protein database that records various protein features. PPI data from HPRD have been applied to thousands of network studies, especially in the field of disease gene discovery.^16^

### 2.2 Human immune‐related gene data sets

All immune‐related genes in *Homo sapiens* were obtained from AmiGo, and 3068 immune genes were obtained based on 651 records.^17^

### 2.3 Guillain‐Barré syndrome (GBS)‐associated genes

We downloaded all GBS‐associated genes from the DisGeNET database, which stores data on human disease‐related genes and variants. We obtained 561 119 gene‐disease associations comprising 20 370 diseases or phenotypes and 17 074 genes.

### 2.4 Immune‐ or GBS‐directed neighbour co‐expressed network construction (IOGDNC)

Firstly, we calculated the Pearson correlation for gene expression between any two gene pairs. Initial gene co‐expression networks were obtained by limiting the expression correlation coefficient (absolute coefficient value > 0.3) and false discovery rate (FDR < 0.05). For the second step, we mapped the PPI network pairs to our co‐expression network and only retained the gene pairs that were common to the PPI network. The final network is a Guillain‐Barré‐specific co‐expression network. Network visualization was performed using Cytoscape. Interaction levels were distinguished by high and modest Pearson correlation coefficients. Most biological networks are scale‐free networks. We therefore checked the power law distribution of our co‐expression network in MATLAB, using the degree distribution data from our network.

### 2.5 Dissecting Guillain‐Barré syndrome and immune‐associated gene features in network

We classified the genes into five groups: GBS (Guillain‐Barré syndrome)‐associated genes, immune genes, GBS‐ and immune‐associated genes, GBS‐ and non‐immune‐associated genes, and other genes. In order to construct the GBS‐directed neighbour co‐expression network (GDNC network), we extracted the first neighbours of GBS genes and GBS‐ and non‐immune‐associated genes from IOGDNC network to get two GBS‐associated networks. The one‐step neighbour network for the GBS and GBS‐ and non‐immune‐associated genes was visualized using Cytoscape, with different node colours representing different gene types. For each gene set, we compared the number of their first neighbours in the network. Next, to analyse the level of interaction with neighbours among different gene sets, we used a cumulative distribution function (CDF) to estimate the degree of the expression correlation for each gene category. Wilcoxon rank‐sum tests were used to compare the co‐expression correlation coefficients between gene set pairs.

### 2.6 Network cluster mining and validation of its classification power

We used GraphWeb tool mine important network clusters that are associated with GBS,^18^ using our constructed co‐expression networks as the input file. Each of the output clusters was plotted using the Cytoscape program. For each cluster, the gene expression data were then used to classify the 14 samples in our study using a consensus clustering method.^19^ This was performed using the ConsensusClusterPlus package in R. We chose the optimum category number determined by the point at which the increase in the area under the cumulative distribution function curve is small. Combining the classification results of the consensus clustering and the real category (disease and control) of the samples, we used a chi‐squared test to investigate the association between the two classification methods. We considered the two type of class to be associated when the test result was significant (*P* < 0.05).

### 2.7 Identifying gene expression patterns in network modules

To explore the genes’ inter‐relationship in each module, we computed the Pearson correlation coefficient S between each gene pair in the module. Correlation heatmaps were plotted in R using the pheatmap package. We also compared the gene expression patterns between GBS and normal control samples in each module. The differentially expressed genes were identified using a *t* test in R, with a significance threshold (*P* value) of 0.05. Finally, we used a hypergeometric test to validate the enrichment between all differentially expressed genes and the differentially expressed genes in all modules.

### 2.8 KEGG pathway enrichment analysis

We performed functional enrichment analysis using the GSEApy package in Python. Briefly, the genes in each module and all modules were tested against each KEGG pathway, respectively. Significant enrichment results (adjusted *P* value < 0.05) were retained for the following analysis.

## 3 RESULTS

### 3.1 Immune‐associated genes are essential in the process of GBS

**Figure 1:**
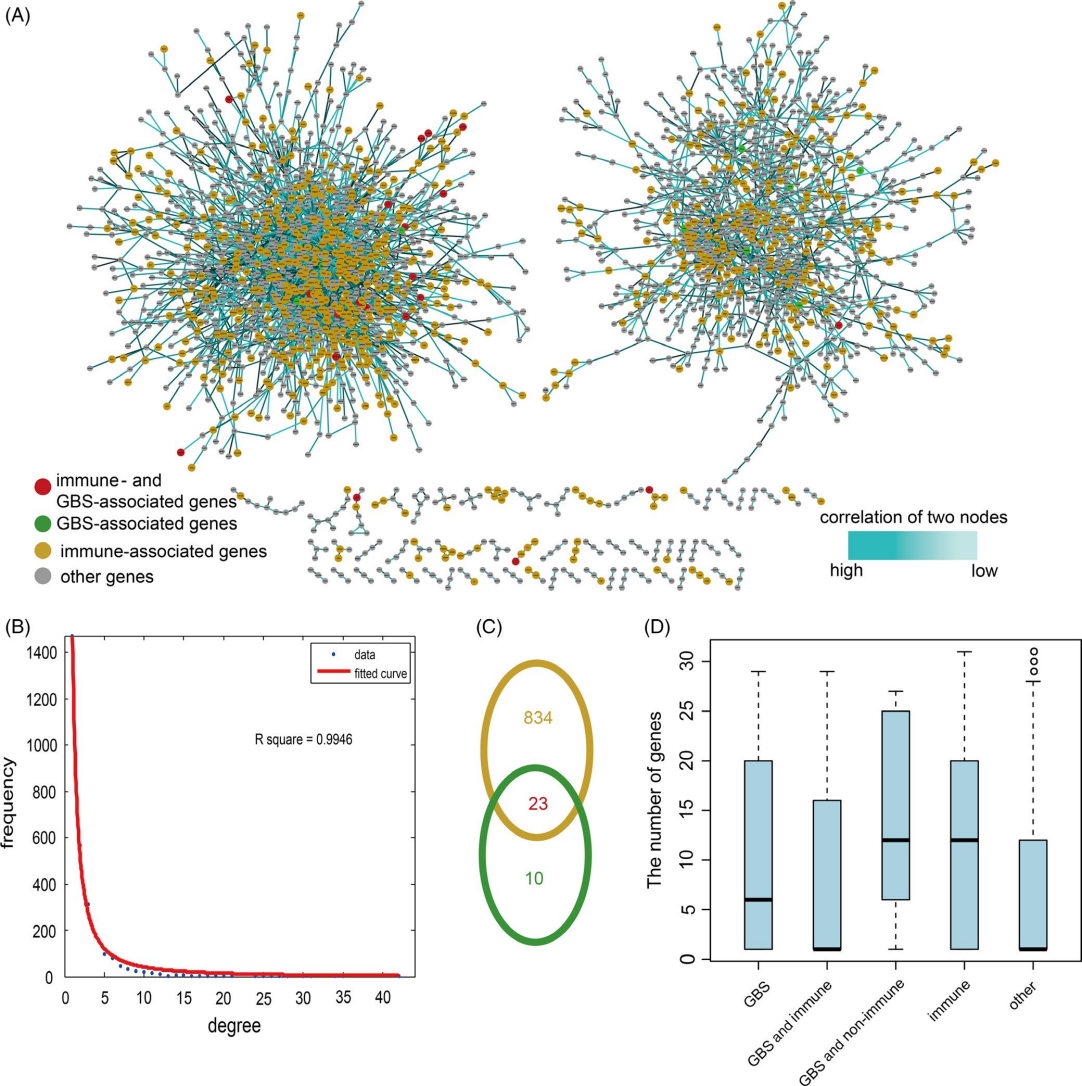
The properties of the immune‐ or GBS‐directed neighbour co‐expressed network (IOGDNC network). A, The global IOGDNC network. B, The degree distribution of all nodes in the IOGDNC network. C, The Venn diagram shows the intersect for GBS‐associated genes and immune‐associated genes. D, The box plots show the degree of different gene types

There were 58 genes common to the immune‐related and GBS‐related gene sets such as *FCRL3*, *CXCL1* and *HPRT1*. Twenty‐four GBS‐specific genes and 2859 immune‐specific genes were also identified. We constructed an immune‐ or GBS‐directed neighbour co‐expressed network (IOGDNC network), which is a subnetwork extracted from the PPI network. The IOGDNC network included 2497 nodes and 3442 edges (Figure 1A). First, the degree distribution of all genes was analysed, with the degree showing the scale‐free distribution (Figure 1B). The R‐squared was 0.9946. There were 23 immune‐ and GBS‐associated genes, 10 GBS‐associated genes and 834 immune‐associated genes in the network (Figure 1C). Next, we compared the degree of different gene types and found GBS‐associated and non‐immune‐associated genes, and immune‐associated genes showed the highest degree (Figure 1D). The top five genes with highest degree were *CASP3*, *PRKCD*, *AKT1*, *EGFR* and *SUMO4,* and three of these were immune‐associated genes. This suggests an essential role for immune‐related genes in the IOGDNC network. Collectively, these results indicate that immune‐related genes could act as essential contributors for GBS.

### 3.2 Immune‐associated genes interact with GBS genes and show stronger co‐expression patterns

**Figure 2:**
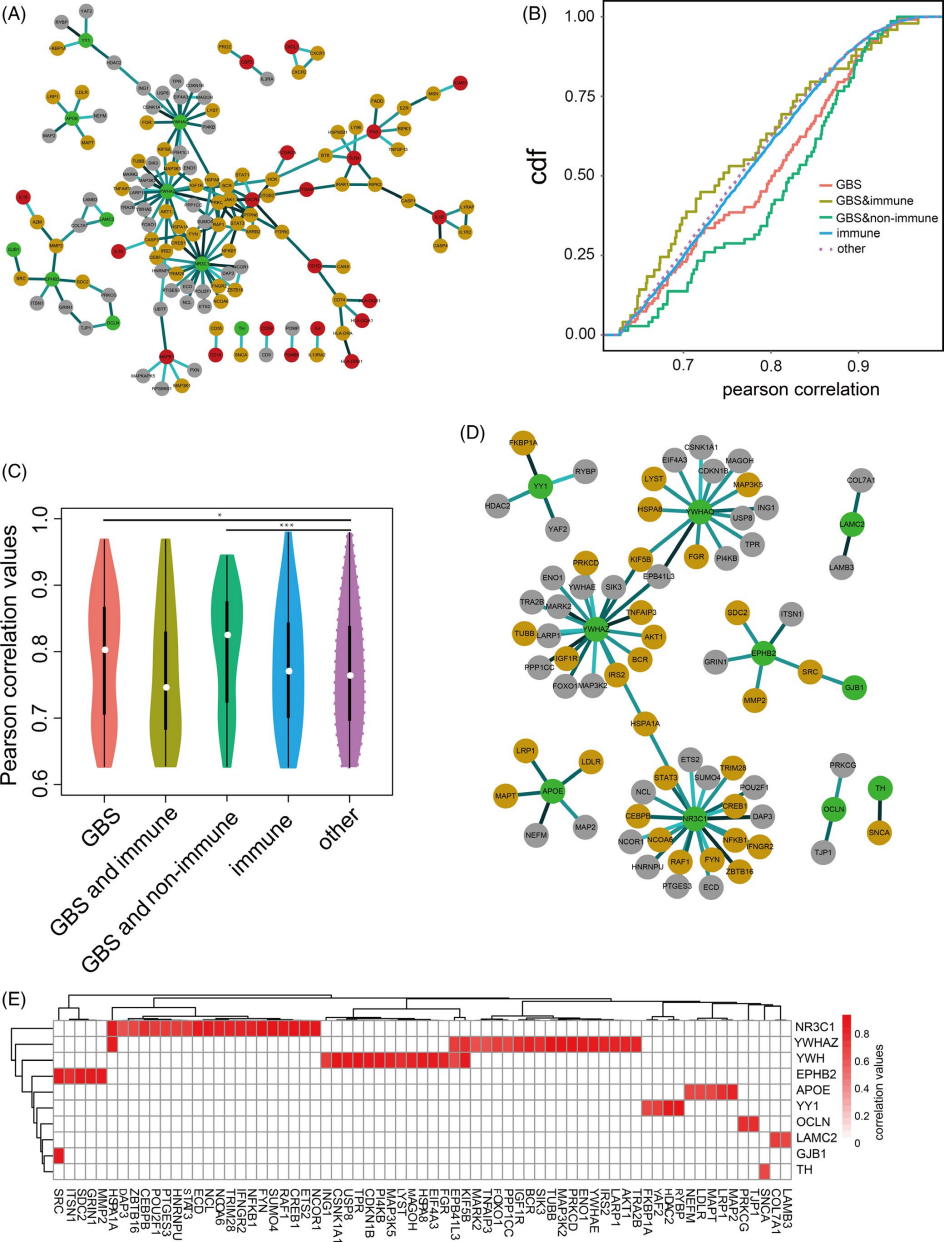
The characteristics of the GBS‐directed neighbour co‐expressed network (GDNC network). A, The global GDNC network. B, The cumulative distribution curves of co‐expression values (Pearson correlations) for diverse gene types. C, The violin plots show the Pearson correlations of different kinds of genes. D, The subnetwork is extracted from the GDNC network which comprises only GBS‐associated genes and their interacting genes. E, The heatmap shows the correlations between GBS‐associated genes and immune‐associated genes

We also constructed a network termed GBS‐directed neighbour co‐expressed network (GDNC network) to further consider the association between immune‐associated and GBS‐associated genes (Figure 2A). The GDNC network is a subnetwork of the IOGDNC network based on extracting GBS‐associated genes and their directly interacting genes. There were 20 GBS‐ and immune‐associated genes, 10 GBS‐specific genes and 63 immune‐specific genes. We further analysed the co‐expression (Pearson correlation) levels of the interactions between different gene types (Figure 2B). The GBM‐e and immune‐associated genes showed the highest co‐expression level with their interacting genes. This result suggests there are strong relationships between immune‐associated genes and GBS‐associated genes based on topological structure and expression patterns. Additionally, we discovered the correlation of expression was significantly distinct among diverse kinds of genes (Figure 2C). A subnetwork only comprising GBS‐associated genes and their directly interacting genes was extracted from the GDNC network (Figure 2D). This network included nine GBS‐associated genes, all of which interacted with immune‐associated genes. These GBS‐associated genes also showed strong correlations with immune‐associated genes (Figure 2E). For example, GBS‐associated gene *NR3C1* was strongly co‐expressed with immune‐associated genes including *STAT3*, *RAF1* and *TRIM28*. These results indicate that there are complex features including topological interactions and expression patterns among the associations between GBS‐associated genes and immune‐associated genes.

### 3.3 Immune‐associated network clusters are special classifiers in GBS

**Figure 3:**
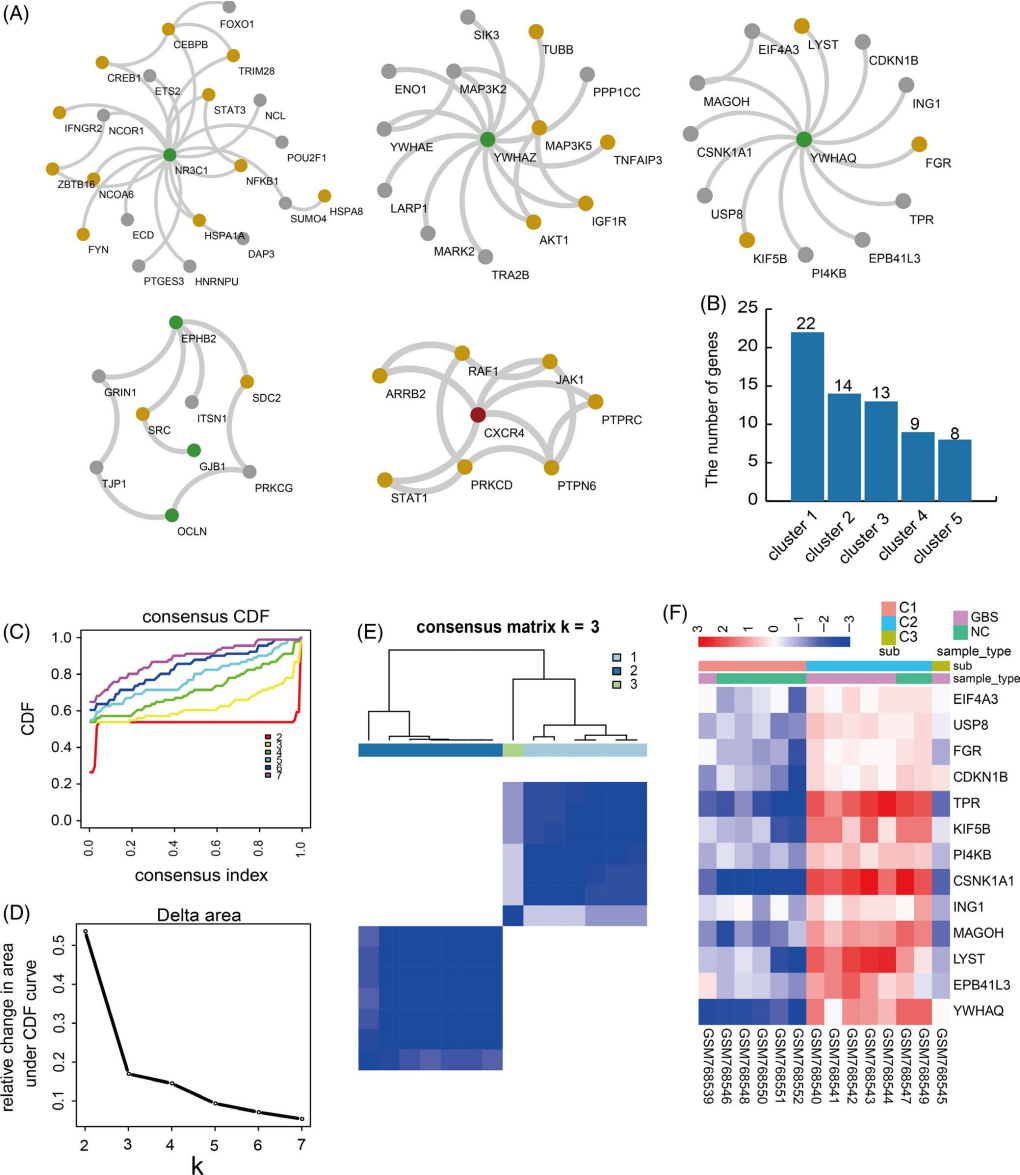
The GBS‐associated clusters and their classifying power evaluation. A, The important module clusters resulted from the IOGDNC network. Different genes are represented by different colours (green: GBS genes; yellow: immune genes; dark red: GBS and immune genes; grey: other genes). B, Numbers of genes in each cluster. C, Cumulative distribution function plot of the consensus index. D, Relative change in area under CDF curve of different group numbers. E, Consensus cluster heatmap of samples. F, The gene expression heatmap, sublabel refers to the group type classified by the consensus cluster method, and sample type refers to the disease status of the samples

We used a public web server, GraphWeb, to identify the vital module clusters from our IOGDNC network. A total of five clusters were identified, comprising several important GBS‐ or immune‐associated genes (Figure 3A). The number of genes in each module was variably distributed (Figure 3B). Most of the GBS‐associated genes in the five module clusters have hub topological features, connecting to immune genes or other genes. This topological characteristic demonstrates an essential role for GBS genes in the gene co‐expression network, along with other key immune genes. For instance, the hub *CXCR4* gene in module 5 is both a GBS‐ and immune‐related gene. Immature plasma cells with low *CXCR4* content have been observed in GBS patients 20; *CXCR4* is also a gene that exerts significant roles in many immune processes, such as myelopoiesis, haematopoiesis and B‐cell lymphopoiesis.^21^, ^22^ STAT1 can regulate multiple immune system reactions, such as cell death and anti‐microbial activities, as biological and medical studies have rapidly developed, the role of STAT1 in tumorigenesis has emerged.^23^ Researchers have demonstrated that JAK1 (Janus‐associated kinase 1) can serve as an inhibitor in the treatment of immune disorders.

Furthermore, we used gene expression values in each module to classify samples by a consensus clustering method. We used a chi‐squared test to validate the classification power of these modules. The test shows that module 3 and module 5 are effective in distinguishing samples (*P* = 0.033 and 0.039 separately). The result of module 3 is displayed (Figure 3C‐F, other modules’ results are displayed in Figure S1). According to the CDF values and relative change in area under curve (AUC) of the CDF plot (Figure 3C,D), we determined that the optimum number of groups is 3 (Materials and methods). Furthermore, each sample group has a consensus expression pattern (Figure 3D,F).

### 3.4 Immune‐ and GBS‐associated genes are related on expression level in clusters

**Figure 4:**
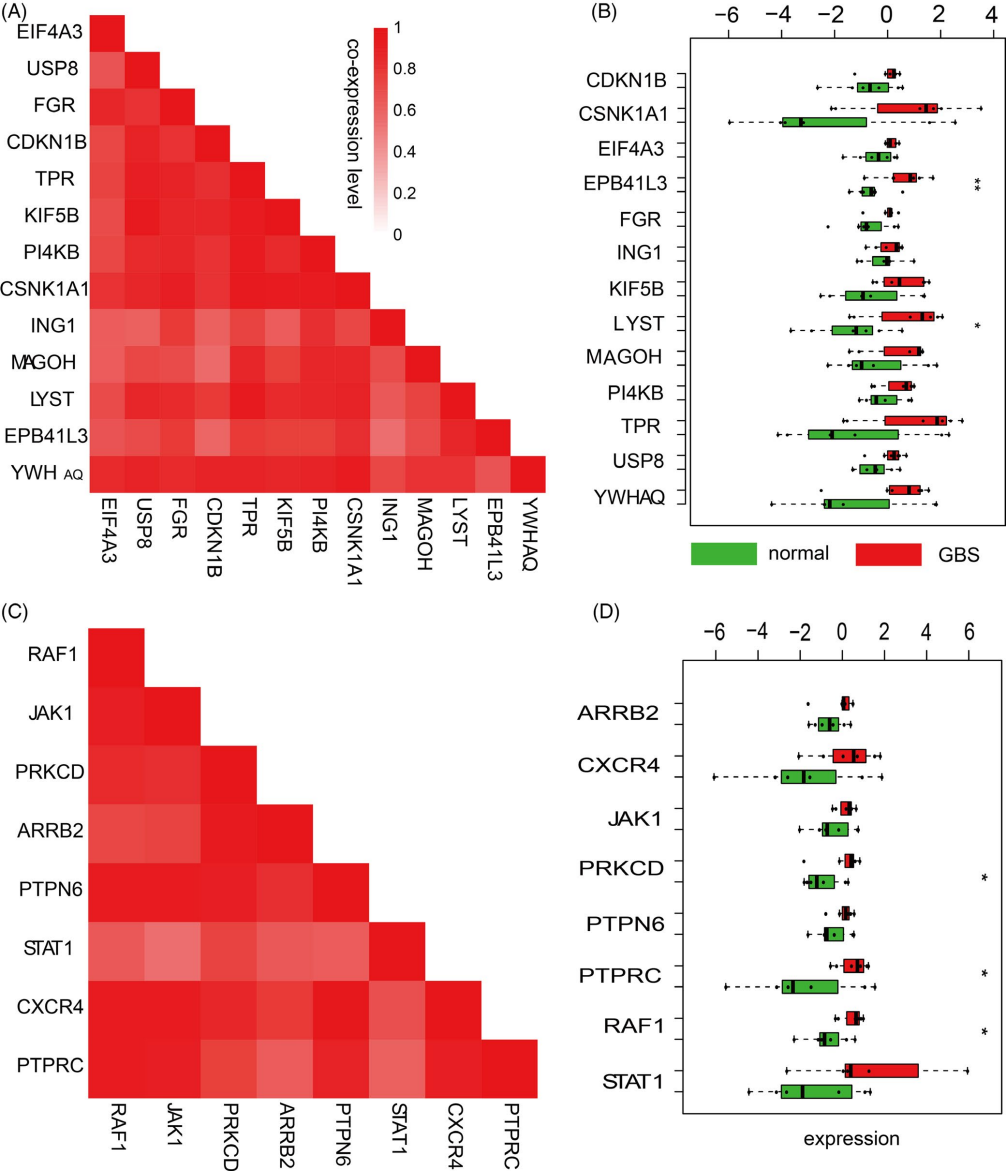
Co‐expression and gene expression feature in the clusters. A,C, The co‐expression heatmap of genes in the third and fourth module cluster. B,D, Gene expression pattern of the third and fourth module, significantly differentially expressed genes have been labelled (* and ** indicate the significance level of 0.05 and 0.01, respectively)

The similarity of the modules based on their co‐expression status was dissected. We found these modules all had a very high co‐expression pattern for any gene pair. The fraction of the co‐expression value >0.5 against all gene pairs reached 90% for all module clusters. In particular, the proportion of the co‐expression values more than 0.7 in each module was 73.6% on average, with the minimum value being 58.2% in the second module and the maximum value being 86.1% in the fourth module. The third and fifth module's co‐expression patterns were very significant (Figure 4A,C, for other modules, see Figure S2).

We also analysed gene expression in the five modules by comparing the expression values for all genes between GBS and normal control samples. Interestingly, in all modules, the gene expression value differed between disease and control samples. For modules 1‐3 and 5, the gene expression in GBS was higher than controls; on the contrary, the module 4 gene expression in normal control samples was higher than GBS samples (Figure 4B,D, and Figure S2). We could infer that these genes have a consistent impact in GBS. In addition, we discovered significantly differentially expressed genes by a *t* test (Figure 4B,D). Although the total number of differentially expressed genes in our module was small, it may be related to the low gene numbers in each module. We assessed whether genes with differential expression patterns between groups were enriched among our module gene sets (Materials and methods). Unsurprisingly, this enrichment was significant (*P* = 0.021).

### 3.5 The functional analyses show the potential significance of immune‐associated clusters in GBS

**Figure 5:**
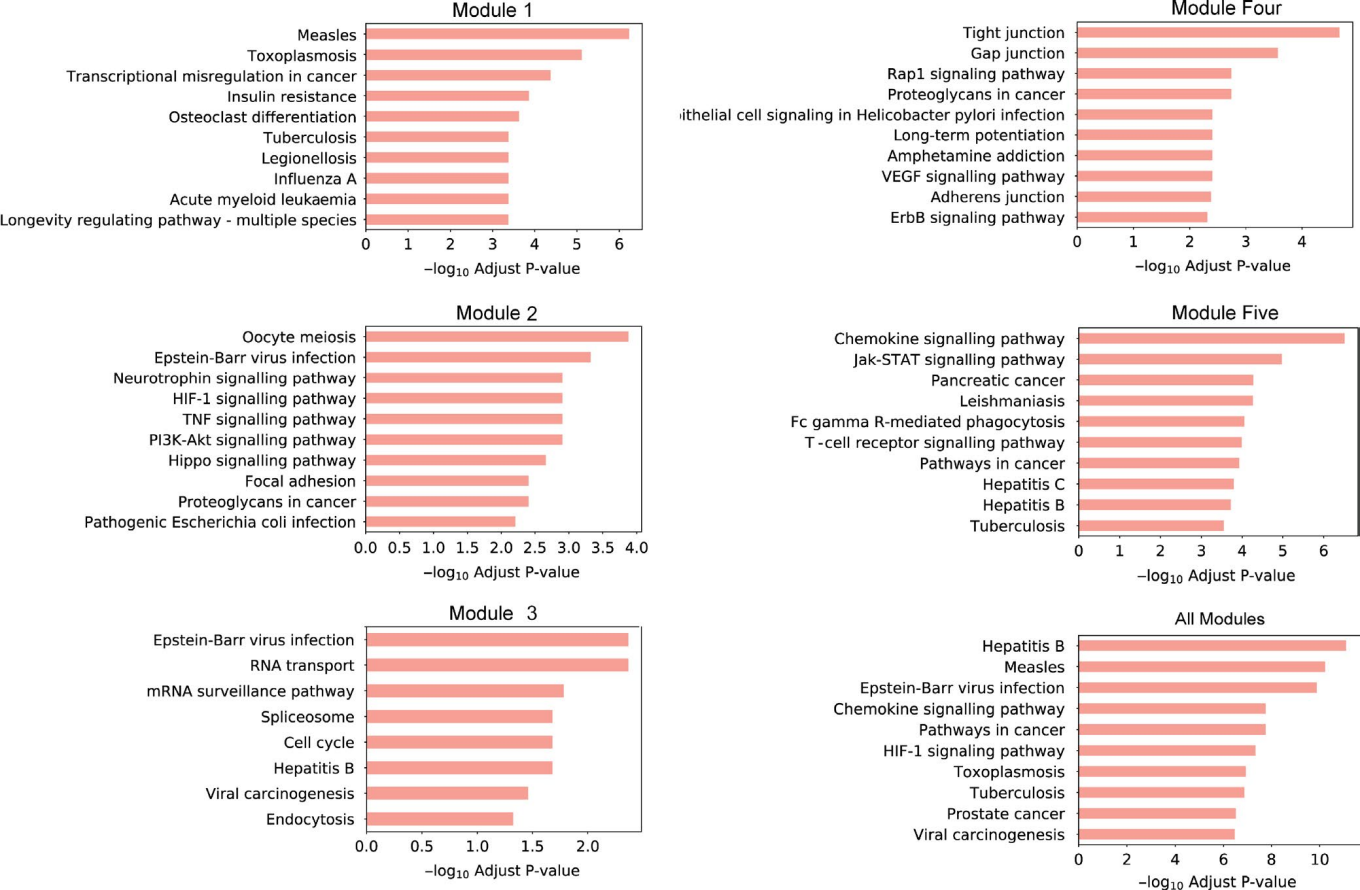
KEGG pathway enrichment analysis in genes. The KEGG enrichment results for the five module genes and all module genes, respectively

**Figure 6:**
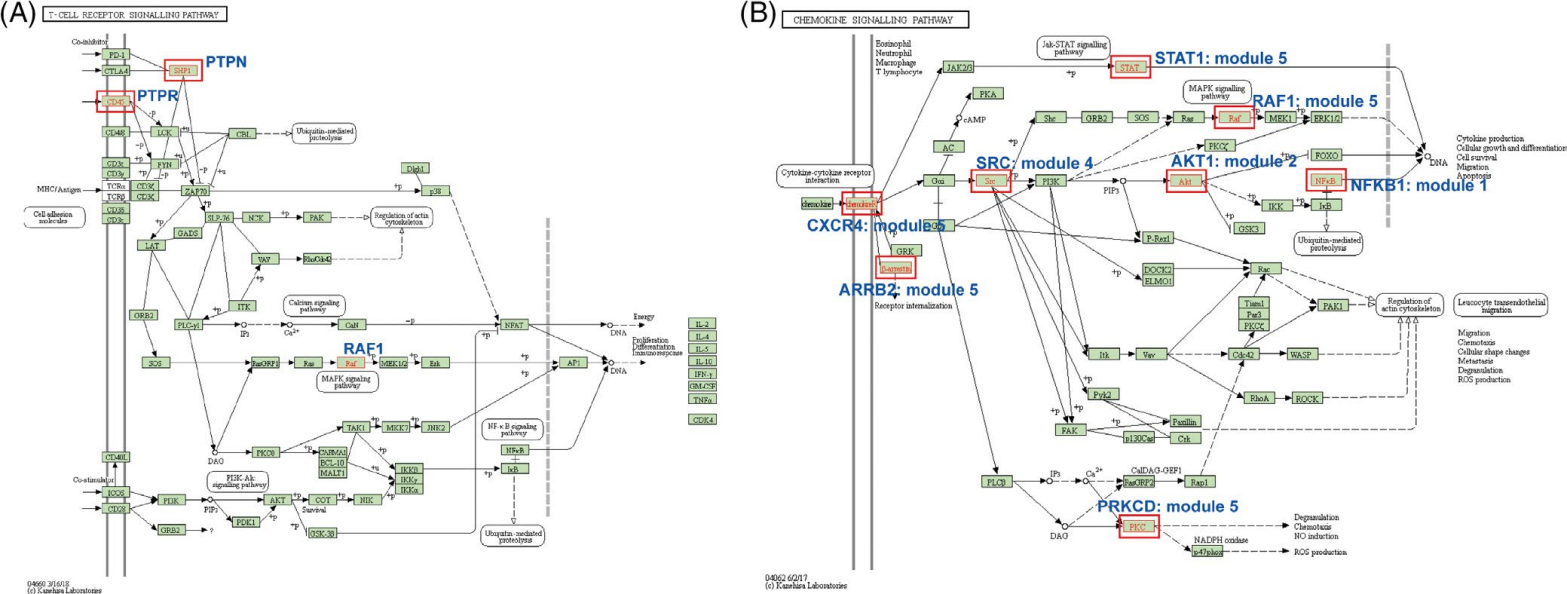
KEGG pathway enrichment analysis in signalling pathways. A, The map of the chemokine signalling pathway. B, The map of the TNF signalling pathway

KEGG pathway enrichment analysis was performed for our module clusters. Because of the low number of module genes, KEGG analysis was also performed for all genes in each module (Figure 5, the whole result pathways are available in Table S1). We obtained several important pathways that are related to the immune system, such as antigen processing and presentation, leucocyte transendothelial migration, chemokine signalling pathway, Fc gamma R‐mediated phagocytosis and T‐cell receptor signalling pathway. When using all module genes to perform pathway enrichment, we find there is a common pathway, chemokine signalling pathway, which is implicated in several biological reactions including immune responses. All module genes except for those in the third module were enriched in this pathway. Early studies identified a role for chemokines in recruiting immune cells to the site of inflammation.^24^ After years of study, new roles of chemokines have been discovered. Chemokines also regulate other cells to impact embryonic development and metastasis. CXCL12 is a well‐known chemokine, and it can bind with CXCR4 (Figure 6A, module 5) to activate downstream signalling reactions.^25^ Moreover, we also identified a significant immune‐related pathway, TNF (tumour necrosis factor) signalling pathway (Figure 6B). The most famous member of the TNF family is TNF‐α (Figure 6B), and its initial function comprises regulating the immune cells. Many studies have demonstrated that the abnormal expression of TNF‐α is implicated in multiple human diseases.^26^, ^27^, ^28^ Researchers had shown that the crosstalk between TNF‐α and TNFR1 have a vital effect in the tumour microenvironment. They also have an important role in tumour progression, and impact metastasis, apoptosis and survival of tumour cells. Dysregulation of module cluster genes might therefore impact GBS status through acting on these immune‐associated pathways, which are potential signatures for GBS.

## 4 DISCUSSION

The current study integrates interaction network and expression profile to explore the functions and mechanisms of immune‐related genes in GBS. It provides novel insights into understanding the development and treatment for GBS. Some effective biomarkers have been identified for GBS in past years. However, the global identification and characterization are lacking. Here, a comprehensive computational approach was developed based on GBS‐related genes and expression profiles to explore the features of GBS‐related immune genes and clusters.

Guillain‐Barré syndrome is an acute immune disease which occurs at nervous system, with a serious prognosis. It has been reported that GBS could be induced by immunization with peripheral‐nerve proteins.^29^ In the current analysis, we also concentrated on the association between immune‐ and GBS‐related genes on gene expression patterns. The immune‐ and GBS‐related gene interaction network and expression profile data were both integrated to study the role of immune genes for GBS. Collectively, all results suggest there are strong associations between immune‐ and GBS‐related genes not only on network structure but also on expression patterns. Some immune‐associated genes may have potential as diagnostic and treatment biomarkers of GBS based on differential expression and functional analyses.

In summary, interaction networks and expression profiles were integrated to reveal novel roles and insights for immune‐related genes in GBS. Immune‐ and GBS‐related genes showed close relations both at the level of network interaction structure and expression patterns. Some immune‐associated clusters were also identified for GBS, and these core clusters comprised special expression features. Specially, gene expression of these modules could distinguish the GBS and normal samples. The functional analyses highlighted the molecular mechanisms of immune‐associated genes in GBS.

## 5 CONCLUSIONS

We constructed a GBS‐directed neighbour co‐expressed network (IOGDNC network) and a GBS‐directed neighbour co‐expressed network (GDNC network) and found an important role of immune and GBS gene in GBS. We identified five clusters from the GDNC network, some of which can serve as biomarkers to distinguish between GBS and control samples. In addition, the genes in these clusters displayed different expression characteristics between GBS and control samples. Furthermore, pathway enrichment analyses clearly illustrate the functions for these genes in each cluster. Our results provide support for these immune‐associated genes as potential therapeutic targets for GBS.

## CONFLICTS OF INTEREST

The authors declare that they have no competing interests.

## AUTHOR CONTRIBUTIONS

ZXM, WLH, SJ and GM conceived and designed the experiments. ZHX, LZJ and ZXM performed the experiment and wrote the manuscript. WJJ, KXT and BCR collected the data. LXY, LJ and BM validated the experiment.
