# Supplementary figures and images for: Inferring immune‐associated signatures based on a co‐expression network in Guillain‐Barré syndrome
Source: Cell Prolif. 2019 May 16;52(4):e12634. doi: 10.1111/cpr.12634 (PMC6668984; doi:10.1111/cpr.12634)

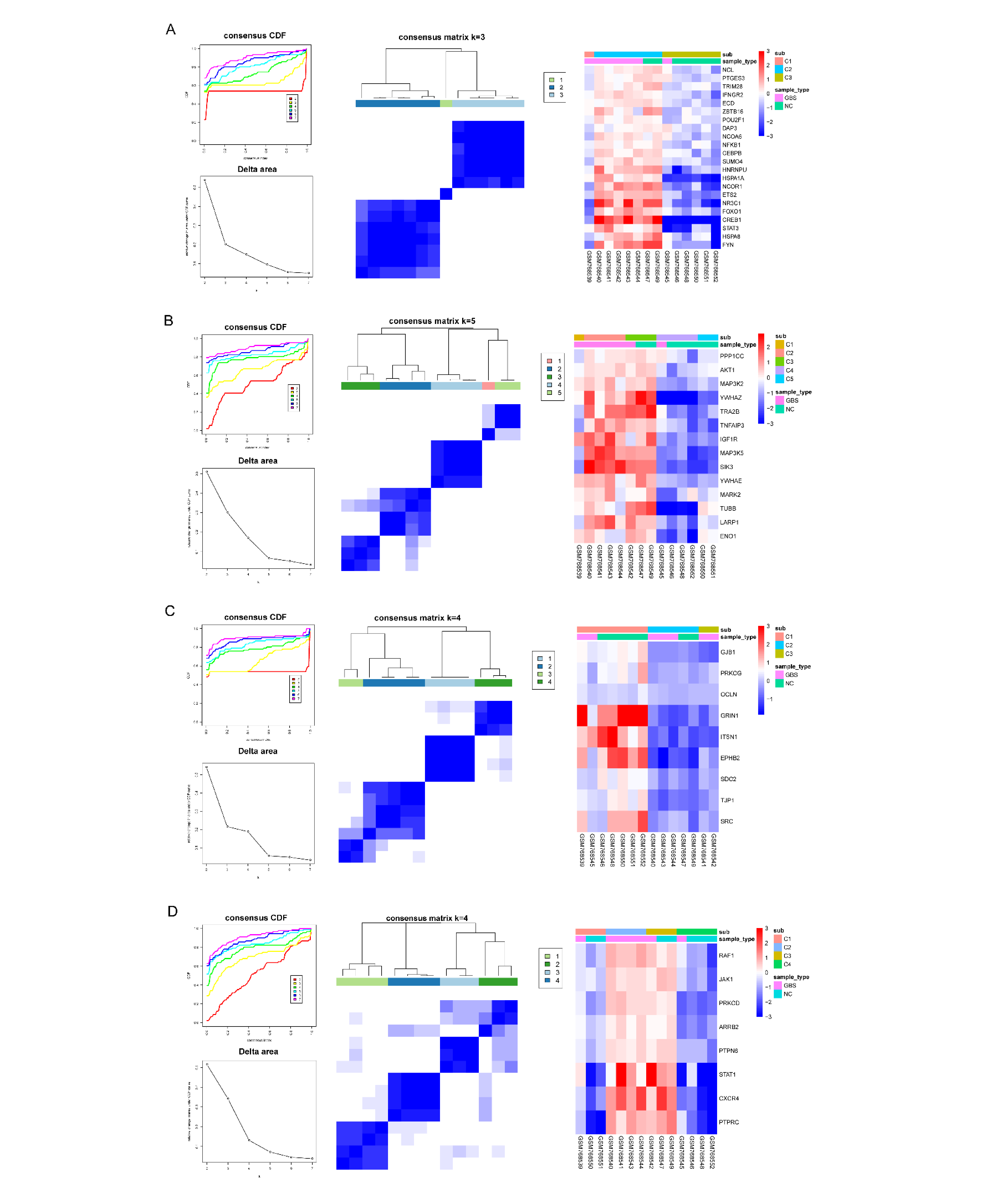

Supplement: Supplementary file 1 [file CPR-52-e12634-s001.tif]

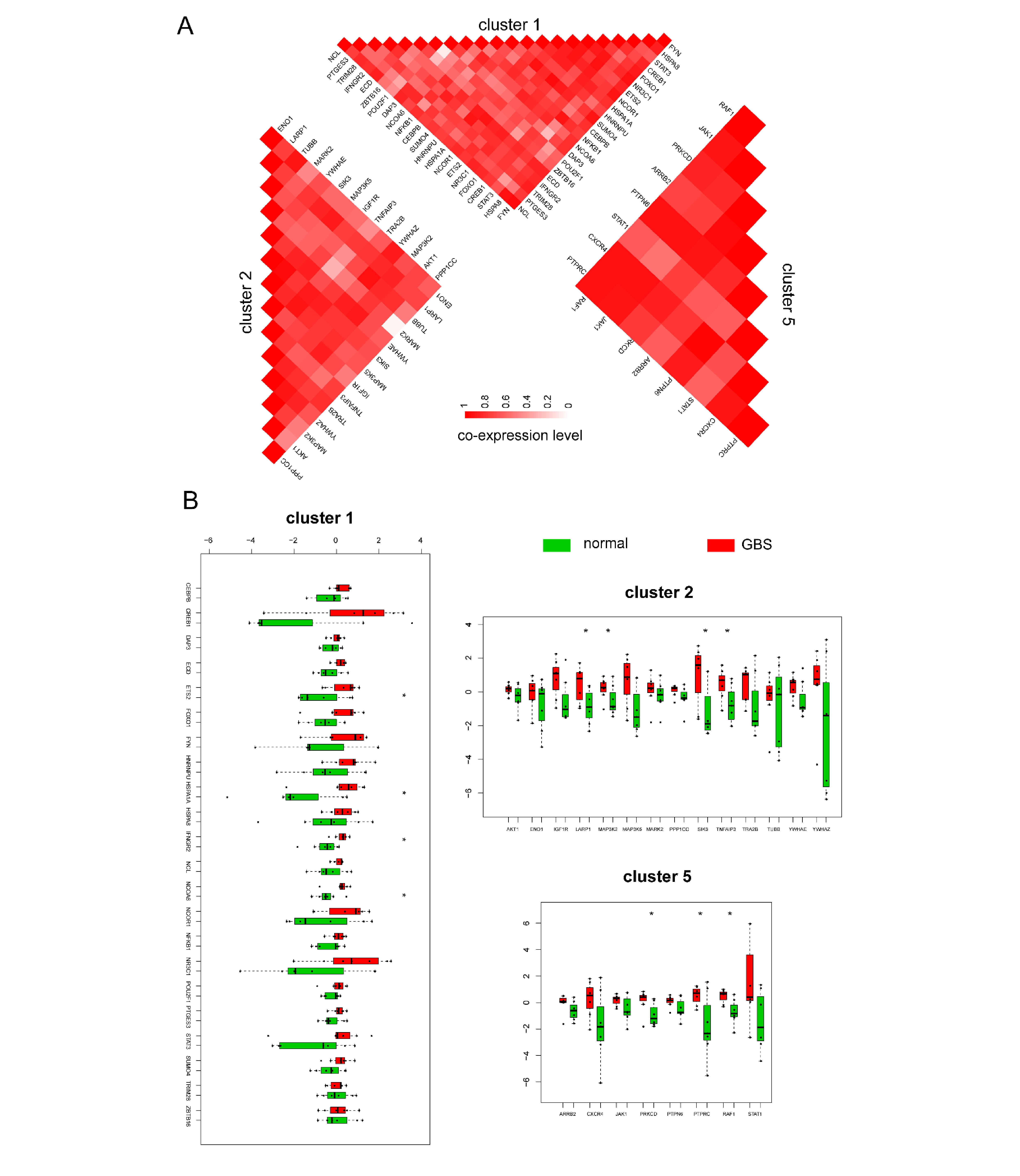

Supplement: Supplementary file 2 [file CPR-52-e12634-s002.tif]
